# “We Should Not Call an Ambulance, Even If We are Very Sick”: Ukrainian Refugee Women’s Experiences in the United States Healthcare System

**DOI:** 10.1007/s10903-025-01710-0

**Published:** 2025-07-04

**Authors:** Yana D. Gepshtein, Jung-Ah Lee, Dawn T. Bounds, Candace W. Burton

**Affiliations:** 1 Sue & Bill Gross School of Nursing, University of California Irvine, 100B Berk Hall, Irvine, CA 92617 USA; 2 School of Nursing, Faculty of Health, York University, 4700 Keele St, Toronto, Ontario M3J 1P3 Canada

**Keywords:** Refugee health, Migrant health, Women health, Barriers for healthcare, Patient trust, Qualitative research

## Abstract

As the number and diversity of refugees worldwide increases, healthcare providers working with these populations face unique challenges. Thus, healthcare providers in host countries have limited understanding of challenges refugees face when using healthcare in host countries. The goal of the study is to achieve an interpretive understanding of women refugees' perspectives on their interactions with healthcare systems and providers, as well as to identify factors and processes that women refugees view as enabling or hindering their access to healthcare. Study participants were refugee women from Ukraine in the US (N = 17), who volunteered to take part in semi-structured interviews about their experiences in the US healthcare. The interviews were transcribed in original languages and analyzed using Charmaz’s constructivist grounded theory methodology, which is based on constant comparison between codes and data exemplars within and between interviews. Three priority theoretical elements were identified: (1) *barriers to care*, which encompassed codes: uncertainty about costs, lack of health insurance, time constraints, difficulties in communication, problems of distance and transportation, and finding a trustworthy provider; (2) *systemic and organizational features that hinder care,* which encompassed codes: the system is confusing, inconsistencies across organizations and providers; limited scope of organizations meant to help refugees; and (3) *processes and factors that do or would alleviate impediments to care,* which encompassed codes: clear and relevant information, getting help from others, addressing patients' concerns, acknowledging patient circumstances. The study emphasizes the importance of continuity of care in refugee health, indicates the culture-bound nature of trust in healthcare providers, and underscores the essential role of non-formal and non-structured support for refugees.

## Background and Conceptual Framework

As an unprecedented number of people worldwide are displaced from their homes, healthcare providers must often take care of patients whose healthcare experiences and knowledge are different from their own. When working with displaced populations, healthcare providers and policymakers face unique challenges [1–5]. Providers may not understand refugees’ legal status, entitlements, health expectations, culture-bound health concerns, past experiences, and living circumstances [2, 3]. At the same time, refugees might struggle to effectively address their health concerns due to economic and transportation difficulties, anxiety in unfamiliar environment, mistrust in health care providers, or limited English proficiency [3, 5].

Research concerning refugee and immigrant health underscore an urgent need for studies of refugees' perspectives on interactions with healthcare systems and the barriers they encounter as a part of these interactions [4]. At the same time, the established health system in the United States largely lacks a framework for healthcare providers to comprehensively understand complex refugee experiences [1, 6–8].

Research on refugee health is complicated by rapidly changing demographics, the socio-cultural diversity of refugee populations, cultural diversity among health care providers, and local differences in health policies towards refugees [6]. Furthermore, refugees' personal stories are often shaped by experiences of trauma and influenced by participants’ views of the researchers as outsiders [1, 9, 10]. In addition, the trustworthiness of the findings can be affected by researchers’ misunderstanding of the culture-bound context of participants’ experiences and by data loss in the translation process [11, 12]. Finally, one of the crucial challenges of refugee research, and cross-cultural research in general, is that researchers and health providers within a healthcare system are rarely aware of the social structures and inherent assumptions of their own social worlds [13, 14].

The goal of the study was to achieve an interpretive understanding of women refugees' perspectives on their interactions with healthcare systems and providers, as well as to identify factors and processes that women refugees view as enabling or hindering their access to healthcare. The central research question was: How do women forcefully displaced by the current war in Ukraine described their interaction with the healthcare system in the United States (U.S.). We use the Constructivist Grounded Theory (CGT) method by Charmaz (2014) to identify processes in provider-patient interactions that are experienced as either impeding or enabling health care for this group of refugees.

## Ukrainian Refugees in the United States

As of December 31, 2023, nearly 6.48 million Ukrainian refugees were documented worldwide [15]. In the United States (US), most Ukrainian nationals escaping war during the first couple of months since February 2022, entered the US through the Southern border under temporary Humanitarian Protection status [16]. An official pathway for Ukrainian citizens seeking protection in the US, the Uniting for Ukraine (U4U) program, was established on April 21, 2022. Under the U4U program, Ukrainian citizens can stay in the US temporarily for two years under Temporary Protection Status (TPS) [17]. According to the US Department of State (2024), as of December 2023, 170,000 from Ukraine have been granted entry into the US [18].

## Impact of War on Healthcare and Displacement in Ukraine

The Ukrainian national health system is mostly funded by the Ministry of Health of Ukraine, the National Health Services of Ukraine, and territorial community funds [19]. Besides the national health system, Ukrainian residents can opt for private health options [19]. Since the beginning of the military conflict, many essential health services in Ukraine have been interrupted: multiple medical facilities had been damaged or destroyed while operations of some medical facilities had been hampered by disruption in supply of energy, medications, or essential equipment [20]. At the same time, thousands of health professionals had been drafted or signed up to volunteer in the army; many left the country or were otherwise internally displaced [20]. Refugees displaced by the Ukraine-Russia war often arrive at their destination with no health records. Many also lack a sufficient supply of essential medications [21].

## Mental Health and Violence Risks among Women Refugees

Devastation of essential medical, social and mental health infrastructure poses a devastating threat among vulnerable populations [22]. Displaced women are at risk of trafficking and sexual violence [21, 23]. Further, displaced women in urgent need of pregnancy termination may find themselves in countries where abortions are illegal [24]. The Center for Reproductive Rights (2023) reported cases where women refugees from Ukraine seeking pregnancy termination had to return to war zones in Ukraine to access care [25].

Despite the relatively welcoming post-migration environment, Ukrainian women refugees are also at high risk for depression, anxiety, and Post Traumatic Stress Disorder [20, 26, 27]. Depression and anxiety are associated with histories of family separation, war-related experiences, unemployment, material struggles, and lack of language skills [28].

## Methods

### Study Design

This study utilized qualitative approaches using individual interviews based on the Constructivist Grounded Theory (CGT) method by Charmaz (2014), which is distinct from other grounded theory methodologies in that it allows researchers to construct a shared meaning while highlighting the nuances and uniqueness of participants’ voices. According to Charmaz, understanding of participant’s experiences is interpretive, as it results from the interactions among researchers, participants, and the data [29]. Shared understanding is generated through constant comparison between codes and data, as well as through iterative discussions and reflections [29].

Transcripts’ translation method was rooted in a constructivist approach to translation posing that language reflects a unique way of constructing or seeing the social world [29, 30]. Accordingly, no translation can accurately represent the language of origin because the spoken language is always loaded with emotions, assumptions, and assignment of meaning [31, 32]. Translation, thus, is a process of constructing a nuanced and, at the same time, shared understanding of the data [31].

The institutional review board (IRB) of the university that the authors affiliated approved the study protocol before the data collection (UCI IRB No.2238). Due to the minimal risk of the nature of the qualitative study, written consent was waived, and study information sheet including the purpose of the study, data collection procedure, data confidentiality, study benefit and cost was provided to participants prior to data collection.

## Data Collection

Study flyers were posted on Ukrainian and Russian language social media sites for Ukrainian immigrants in the US. To access such sites, the PI contacted site moderators and described the study’s process and purpose. The PI received twenty replies from potential participants. Nineteen of these agreed to proceed after they reviewed study information in Ukrainian, Russian, or English, and were invited for semi-structured virtual 60-90-minute-long interviews (with or without camera) in Ukrainian or Russian. Two of the interviews were not completed due to unexpected scheduling issues. According to CGT method [29], data analysis started at the time of data collection. The interviews were collected in March and April of 2023 until theoretical data saturation was reached, when researchers when researchers did not find any further novel characteristics or properties of the emerging categories [29].

The interviews were conducted by the PI, who is a native Russian speaker, a certified medical translator (Russia-English), and is fluent in Ukrainian as well as English. The participants were asked about their health, experiences with healthcare organizations, and self-care through their lives, displacement, and resettlement. Pertinent demographic data were collected during the interviews (Table 1). The interviews were audio recorded, transcribed, and de-identified. After the interview, participants received an incentive of $30 as e-gift card.

**Table 1:** Demographic characteristics of the study sample

Variables	Frequency
Age	
20–29	2
30–39	6
40–49	5
50–59	2
60–80	1
Not given	1
Marital Status	
Married with children (spouse in US)	4
Married with children (spouse in Ukraine)	7
Married no young children	1
Single	4
Widow	1
Educational Level	
Postgraduate	9
Professional: medical doctor/pharmacy	3
Some college	1
Not given	4
Place of residence	
Western region	6
Southeastern region	3
Midwestern region	4
Northeastern region	1
South central region	2
Other	1
Political status	
TPS	14
Seeking political asylum	1
Uncertain	1
Tourist Visa	1

## Data Analyses

Interviews were transcribed in their original language using an automatic transcription tool, Whisper, downloaded from an open AI platform [33]. Once the program was downloaded, data were audio transcribed in the original language. Any errors in the textual output were revised and manually corrected when compared to the recorded audio.

The transcripts were first coded in the language of origin by the PI using qualitative data analysis software Atlas.ti, version 24. The coding consisted of two stages: initial coding and focused coding. The goal of initial coding was to familiarize researchers with the data by assigning names (codes) to each segment of the data. The goal of focused coding was to (1) identify patterns within both coding and in data, (2) organize and synthesize initial codes, (3) identify the most frequent and significant codes relevant for the research question, and (4) construct code families and theoretical elements.

To preserve nuances of participants’ voice and expressions, initial coding was done based on the language of origin while simultaneously reading through the transcripts and listening to audio recordings of the interviews while paying attention to emphasis and intonation [31]. Initial coding was conducted using line-by-line and in-vivo methods, which involved using participants' own terms and phrases [31]. These initial codes, drafted in English by PI, were refined through an iterative process of constant comparison between codes and data exemplars within and between interviews, and through iterative discussions among the authors [31]. During the focused coding stage, the codes were analyzed for redundancy and grouped into categories for synthesis by the team of co-authors. This synthesis was achieved through through the process of constant comparison between codes, data, and the emerging categories.

## Results

### Sample Description

The participants in this study (*N *= 17), identified as women, had moved to the United States (US) because of the current Russia-Ukraine war, spoke English, Russian, or Ukrainian, and volunteered to participate in a one-on-one interview about their experience of interaction with the health care system in the US.

We conducted sixteen interviews: two participants spoke Ukrainian, while the rest spoke Russian. Two participants requested and were granted a joint interview. Sixteen participants lived in the US, and one had left the US by the time of the interview due to the inability to get health coverage. The interview with this participant was conducted through Zoom while she was residing in a third country. Detailed description of participants’ demographic characteristics is presented in Table 1.

## Results of Data Analysis

We identified three priority theoretical elements: *barriers to care, systemic and organizational features that hinder care, and processes and factors that do or would alleviate impediments to care*. These three theoretical elements, coding themes, and exemplars are summarized in Table 2.

**Table 2:** Summary of study results

Theoretical elements	Code families	Exemplars
Barriers to care	*Uncertainty about cost*	*I am afraid to get sick. It might end up being some inadequate bill.* *They bill for everything, because of this it is scary to go* *It is a strange system, the way they calculate bills. You don’t know what to expect, this is scary and confusing* *The system is complex and what is frightening is very expensive medicine.* *They say that it is going to be without pay but ends up not being covered or covered only in part* *(while in another country) I feel safe…and emotionally it is easier, I can go to a doctorand I. would not have to (be billed) $140,000 if my child had appendicitis*
	Lack of health insurance	*According to the program, we were expected to get health insurance. But they denied me three times* *They denied my (application), (they told me) neither you nor your child can prove your residency* *Whatever is written on the sites for refugees, it does not work (here). If you are not pregnant or have children, you don’t have health insurance* *A worker advised to go to a different state. Some states give insurance right away while others… have problems* *(chooses not to apply for benefits to keep an option leaving the US and coming back if needed): I (would not be able to) leave (or) I… return… for now I don’t apply for benefits*
	Time constraints	*I spent hours in a pharmacy and did not receive my medications.* *They did not have any available appointment and asked me to call some other time. I am not going to call anymore; I spend two hours of these phone calls.* *To sign up for a doctor I must travel 30-40 minutes each direction.* *There is a fear of getting sick. If something happens you must wait for nearly two months.*
	Difficulties in communication	*Every day I get a pile of mail. We don’t know what it is about.* *It had been a week as I keep trying to file a (health insurance) application. Once a reach some point, it shows error. I don’t know if I can (do it)* *To sign up for a doctor I must go there each time. It is still difficult for me to explain over the phone* *It is still difficult for me to explain over the phone. At times I cannot find common (understanding). I start worrying. I can, in principle, explain with words, but they start asking additional questions. I start getting nervous* *here it is very popular (to send) text messages. For us it is Viber, WhatsApp....we don't use SMS on the phone* *I live in a small town. Here it is impossible to find an interpreter.* *It would be nice to speak to a doctor without an interpreter. Otherwise, it is too narrow. It would be nice to ask for some advice* *My interpreter did not interpret correctly. Also, it was a delicate problem. I was uncomfortable talking through a (male) interpreter*
	*Problems of distance and transportation*	*She is a single mom. She cannot buy a car. Her resources are limited. Thus, she cannot have transportation* *I have a very limited choice of clinics accessible by foot. But the clinics that (take insurance) and accept new patients are on the other side of the city* *Providing transportation would help immensely. I must take a bus each time I go to a doctor, and I am very weak* *During weekday, I don’t have transportation. Everyone works and cannot give me a ride* *If someone could help me arrange transportation. I don’t speak any English*
	Finding a trustworthy provider	*I did not find a doctor for myself. I don't know how to get a doctor. And we understand that doctors can be different* *The problem also is that in Ukraine I would not go to just any doctor, there are many doctors who, I cannot say not competent, I just don't trust them* *I was advice a pediatric association, where we, recommended a doctor, we signed up and came while understanding who we go to, and understanding the situation*
Systemic and organizational features that hinder care	*The system is confusing*	*I still don’t understand the mechanism. It is difficult because of helplessness because you don’t understand* *It would help, at least one episode on YouTube explaining about insurance cards, how they work, what are they used for, how do you receive them* *I asked on a forum which doctor takes my insurance: and there is a silence* *It is very confusing. Many Americans don’t understand this system completely.* *It is a strange system, especially the way thy calculate bills. You don’t know what to expect. It is scary to go* *Everything is unknown. I am still figuring it out. It is bad that there is nobody here who could explain*
	Inconsistencies across organizations and providers	*Whenever we apply for help, they always see our address, but the doctor we got for my child and the doctor they assigned to me are very far away* *for 1.5 hours they were figuring out what is that with my insurance, and why I had an insurance and now it is different insurance* *I showed them (refugee assistance) a letter: here, it stated that I was a refugee from Ukraine. First, they did not know anything (about it)* *they tell me they need referral from a doctor. I call to the clinic, asked to send fax. Now they say, I needed some kind of pediatrician note, note from pediatrician. Even in my clinic they do not understand what I want* *It is understandable, how can I go (to a doctor) with fever, when I cannot get up, cannot eat, how can I go get medications? So yesterday I went to get medications. We were standing there and did not get medications…(They) told me that. a doctor did not have contract with (insurance)... But here is my question: what does it have to do with me?*
	*Limited scope of organizations that are set to help refugees.*	*A Refugee Center, which helped me to apply for health insurance last year, notified me that they already helped me and that from now on I have to apply myself.* *I try not to go there if it's not absolutely necessary (extra time lit.). Because...my case manager, she is always so nervous, it seems, it is because they have so many people* *The refugee services helped me a lot at the beginning: they not only helped me with Medicaid but also with money, and that helped me with housing. But, again, I started getting it only after 7-8 months and I was working hard to get it. It was horrible. I was constantly asking for help. At the end, I gave up because I was uncomfortable* *there are organizations that are intermediates between services for refugees and refugees...These organizations are listed on a Refugee Services site. But again, it is extremely difficult to reach these organizations*
	Clear and relevant information	*I could not find clear and official information) for people who came, and a clear system* *If at least there was an episode on YouTube, which would explain that there are those objects, those are Medicaid cards, this, that is what they are used for, that is what they are needed for* *They gave him (the husband) disability status, but we don't know how it works* *I would like to have a unified system, clearly unified system. For example, to know where I go to get insurance, where do I go if I have an insurance, or where do I go if I don’t have insurance (where it is less expensive)* *you need to know where you need to call and (what) to say, a specific situation*
Processes and factors that do or would alleviate impediments of care	Informal help from others	*sponsors. We met good people, and they helped us a lot. And with my husband. To drive us to places* *Yes. I was helped a lot. I turned to a (non-profit) ... They submitted my documents… Back then I lived with an American family, and at first, they also helped me. They looked through my documents, they explained what was needed and what information was needed. I had personal assistance at that non-profit* *Now there are Charities that help during the first year, seriously morally and financially. Perhaps I just did not know about them*
	Addressing patient’s concern	*I liked the doctor very much. She was not pumping things up… I told her I was concerned about respiratory obstructions. I was worried if there is something here to do. She told me that she would prescribe something, a preventive measure* *And the doctor was very good, very good. I showed him my prescription from my Ukrainian doctor, and he said: "that was exactly what I was thinking. and he gave me muscle relaxants. It helped me to get through until my appointment* *I came to you with one problem, and you diagnosed (me) with different problems. It was not planned, and no solution for any of those (my problems) was offered*
	*Acknowledging patient’s circumstances*	*..before my first visit to physiotherapy, or when I already came in, they all were: “hello, how are you? Are you from Ukraine? How are you? I started crying right there. I never cry in front of people, but I just sat and cried because they just asked me " how are you?"* *There is a school nurse in my daughter's school… All that stress and language. It was later that I understood that she found that nurse, by chance. (The nurse) is so good and kind, (My daughter) started going to that nurse's office every day...on any occasion and asked to call me and talk to me...I understood that here a nurse is more than just a nurse...She can evaluate if the situation is urgent, or important, or needs a medical doctor or not. All this is great, such as the great quality of a school nurse* *There is one good thing about that clinic. In the reception, there is our woman from Ukraine, A. I call her A (an endearing form of a name). But she is very pleasant. It is natural, we speak the same language*

The most common themes mentioned by participants were those coded as: (1) time constraints (mentioned in 13 interviews), (2) inconsistencies across organizations and providers (10 interviews), (3) uncertainty about cost (9 interviews), (4) getting help from others (9 interviews).

### Barriers to Care

The theoretical element *barriers to care* included six code families capturing descriptions of processes and factors that influenced participants’ decision to postpone or avoid seeking care. Such code families were: (1) uncertainty about costs, (2) lack of health insurance, (3) time constraints, (4) difficulties in communication, (5) problems of distance and transportation, and (6) finding a trustworthy provider.

Participants believed that the cost of healthcare in the US was “disproportionally high,” “inadequate,” or “scary,” and they described the billing process as confusing and uncertain (*uncertainty about costs* code family). At the same time, several participants in the study failed to receive health insurance despite it being guaranteed by their TPS. One of the participants chose not to apply for TPS and health insurance because the status would not allow her to leave the US and visit her family in Ukraine if needed (*lack of health insurance* code family).

The *time constraints* code family encompassed participants' statements and descriptions about how long it took them to access or effectively utilize healthcare services. This family of codes is related to two other families, *difficulties in communication* and *problems of distance and transportation,* as they included experiences that added to participants’ emotional burden and sense of powerlessness. For example, participants explained that it was “useless” to keep trying to contact a health organization via telephone and “impossible” to get to a clinic they were assigned to without a personal car. At times, such experiences prompted participants to abandon their efforts to seek care. Ultimately, participants explained that they felt the system was founded on the “worthlessness” of the time invested by its users.

Finally, the last code family in this category comprised participants’ statements that they were hesitant about accepting care from an arbitrarily assigned provider. Participants stated that in Ukraine there were usually attending doctors they knew well or whom they had learned about in advance. Participants emphasized that it was vital for them to know in advance “who do (they) go to” and “what to expect.”

### Systemic and Organizational Features that Hinder Care

The theoretical element “*systemic and organizational features that hinder care”* included three theoretical code families. These encompass participants’ reflections on organizational or systemic features that make use of health care systems difficult or create barriers to care. The code families were: (1) *the system is confusing*, (2) *inconsistencies across organizations and providers*; (3) *limited scope of organizations meant to help refugees*.

The first code family in this category included statements supporting participants’ view of the health care system in the US as “complicated,” “confusing,” and difficult to comprehend, even by US citizens. The second code family included examples of lack of coherence and coordination between different elements of the system, which complicated participants’ utilization. Thus, different elements of the healthcare system, such as insurance companies, medical providers, and pharmacies, did not communicate or align efficiently, causing participants frustration and delays in care. As a result of this disconnect, medical referrals were not sent in a timely manner, patients could not receive essential medications, and patients could not access medical offices to which they were assigned by their health insurance (Table 2). The third code family within this category comprises participants' descriptions of the limited capacity of government refugee assistance agencies to offer comprehensive support for refugee needs (see Table 2).

### Processes and Factors that do or Would Alleviate Impediments of Care

The theoretical element “processes and factors that alleviate barriers to care” encompassed code families wherein participants described factors and processes that either currently or potentially helped them in overcoming barriers to care. Included code families were: (1) *clear and relevant information*, (2) *getting help from others*, (3) *addressing patients' concerns*, 4) *acknowledging patient circumstances*.

Participants explained that they wanted clear and official information on how the system works. Such information, for example, could include simple “algorithms,” “rules,” and guidance about what to do in specific cases, such as health emergencies (*clear and relevant information* code family). Reflecting on their interactions with care providers, the participants viewed positively those interactions, in which the provider addressed their relevant questions and concerns and avoided engaging in non-relevant explanations (*clear and relevant information* code family).

The code family *getting help from others* encapsulated statements of appreciation and gratitude toward informal help that participants received from people or organizations such as doctors, volunteers, attorneys, sponsors, or nurses. Such informal support from people in the US made a difference in their experience throughout their journey.

One participant expressed her gratitude towards an attorney, who met them at the Mexican border and helped with acquiring permission to enter the US with TPS status: “We tried (to cross the border) several times but could not. We met a US attorney who volunteered to help us free of charge, and, as a result, we received a humanitarian status entry permit.” A different participant expressed gratitude towards a volunteer who arranged a place to stay during their first months in the US: “If we had not met her, I don’t know what would happen to us. She found people who gave us shelter for the first time.” Yet another participant mentioned a local dentist who waived her child’s visit fee, while one other expressed gratitude to the health providers, who were like “angels” trying to help her during her child’ visit in an emergency room (Table 2).

Finally, the *acknowledging patient circumstances* code family encompassed instances when participants felt that the care they received was based on an understanding of their circumstances and humanity (Table 2). One participant explained: “I always pay attention to the human factor.” In this context, the ‘human factor’ was described as a doctor’s ability to listen to a patient and adapt clinical practice to the patient's needs: “The doctor who manages my children always listens and says, “Okay, I’ll think about it and check…”.

In another example, a participant described consistent emotional support provided to her daughter by a school nurse. As this participant explained: “All that stress and language (difficulties). (My daughter) started going to that nurse's office every day…I understood that here a nurse is more than just a nurse…She can evaluate if the situation is urgent, or important, or needs a medical doctor or not…such as the great quality of a school nurse.”

## Discussion

This study employed CGT methodology to understand how participants interpreted their interactions with healthcare providers, administrators, and refugee assistance agents in the US. The CGT approach allowed researchers to investigate phenomena by engaging with participants, data, and colleagues, with the results emerging through a collaborative effort [29, 34]. This approach enabled researchers to uncover how participants make sense of societal patterns and processes as they unfold within specific temporal, geographical, and social contexts [34].

The results of this study illustrate how experiences of refugee women from Ukraine are shaped by multifaceted interactions of legal, organizational, interpersonal, and systemic barriers. The findings add to research that identifies common barriers to care in refugee populations, such as problems of transportation, difficulties with communication, limited knowledge of the system, and unreliable health care coverage [2, 4, 35, 36]. At the same time, the study adds to the literature in that our findings underscore the urgent need for well-coordinated and comprehensive care for refugees [21, 37–39].

Our participants’ perception of the system as confusing resulted from personal and vicarious experiences of systemic inconsistencies and lack of collaboration between organizations and providers. The importance of coordination between elements of health systems in refugee care delivery had been emphasized in multiple studies [35]. Our findings indicate that lack of coordination between healthcare entities, such as refugee assistance services, health insurance, and health providers, produce and propagate barriers to healthcare, such as the risk of miscommunication, fear, and uncertainty regarding health interactions, failure to obtain health insurance, and a long-time access to care.

As revealed in our study, participants struggled to find answers to basic practical questions about the use of healthcare system in the U.S. and viewed it as incomprehensible, confusing, and unpredictable. This was despite the fact that over 70% of participants had a graduate-level education, and at least 75% had some college education or degree (Table 1). Previous research demonstrated that limited linguistic skills and perception of the healthcare system as complicated hinder utilization of healthcare among migrant populations [40]. At the same time, research has shown that a combination of health literacy and language proficiency correlates with favorable health outcomes in migrant populations [41].

Researchers also argue that, for health information to be helpful, it needs to be individualized, and the information needs of refugees and immigrants must be addressed in the context of their experiences [38]. In this study, participants described their health experiences as positive, when the information they were provided addressed their specific health concerns. Participants also valued health care providers and staff who acknowledged refugees’ condition and demonstrated understanding of refugee experiences.

Besides issues of knowledge and collaboration, two other common themes in research on refugee health are difficulties in communication and mistrust of healthcare providers [35]. During clinical visits, communication between patients and providers can be mediated through interpreting services. Our results are congruent with previous studies that demonstrated conflicting attitudes towards interpreted medicated clinical visits: although patients are commonly grateful for translation services, they often express concern that translation may be inaccurate, and that information is at times omitted through the process [42]. At times, interpreters also are viewed as disturbing the dynamic of the visit and may be perceived as assigning judgment to a patient [42].

Similar to previous research, our participants described trust as a crucial factor when deciding to see a healthcare provider [35]. Healthcare providers working with refugees may not understand the myriad historical and cultural factors influencing patients' confidence in and comfort with providers. Literature about how skills and habits developed through navigating healthcare in refugees' home countries shape their health behaviors in a new social environment, and how these skills and habits are affected by migration and the post-migration environment is generally scant.

In the context of the Ukrainian population, distrust of healthcare providers may also be partially inherited from the post-Soviet past. Although care was available and free of charge, it was part of a centralized, institutionalized system under an authoritarian regime [43–46]. As such, the system was designed to reinforce the ideological values of the time, intrude upon the private lives of its citizens, and even suppress undesired behaviors and ideas [43–45]. In clinical practice, this disregard for the individual, coupled with the view of women's bodies as tools for communal interest, manifested in widespread mistreatment and abuse of women during gynecological examinations and childbirth [43–46].

Finally, participants in our study offered valuable insight into helpful processes that mediate barriers and improve care through therapeutic communication with healthcare providers and community involvement. Previous research suggests that trust between refugees and healthcare providers can be established during visits by respecting patients' knowledge and priorities, using language that patients understand, and providing a safe patient environment [47, 48]. Findings of an interpretive scoping review by Radl-Karimi et al. (2020) suggest that experiences of health can be co-produced through therapeutic interaction between immigrants and healthcare providers. Research also demonstrated that the behaviors of providers conducive to therapeutic relationships with the patient include acknowledgement of the patient's circumstances and empathy [47].

Our participants explained that to address gaps in their care and to find their ways within a complex and fractionated healthcare system, they often relied on informal help from volunteers and peer support. International scholarship demonstrates that when accommodating large numbers of refugees in the health care system, it is necessary to involve multiple organizations from civil society and to design flexible culture-sensitive settings of care [49].

This study has some limitations. First, it addresses the experiences of a small group of educated women from a low-middle-income country. Moreover, the study revealed a variety of local experiences of refugees within the US, and those experiences were greatly influenced by local social, political, and geographic factors. More research should be done explicitly focusing on the locally embedded experiences of refugees. Furthermore, the study also does not consider the socio-political and economic circumstances in which these experiences unfold, and thus our findings cannot not account for structural and organizational policies that may also have shaped participants' experiences. Finally, the study could also gain more depth by including interviews with health care providers, administrators, and refugee assistance agents.

## Contribution to the Literature

This study adds to the literature emphasizing the importance of continuity of care in refugee health and coordination between system elements. Our analysis also indicated the culture-bound nature of trust in healthcare providers and added support for the argument that to improve care for refugees, it is imperative to understand refugee experiences and health behaviors in the context of their pre-migration, migration, and post-migration circumstances. Furthermore, the study underscores the essential role of non-formal and non-structured support for refugees as instrumental in the overall refugee experience. Further research should explore specific features of this non-formal support that enable refugee care, which can be used as exemplary when designing health programs for refugees.

Finally, the literature on applications of CGT in cross-cultural studies is still limited. Data are limited on using different translation methods in cross-cultural qualitative studies [30–32]. The proposed study will thus also contribute to this scholarship [31, 32].

## Data Availability

The datasets generated during the current study are not publicly available because all study participants are refugees from a military conflict, and the use of the data must be carefully monitored to protect participants’ safety. Excerpts from data are available from the corresponding author on reasonable request.
